# Immunological and reproductive effects of anthropized environments in *Leptodactylus fuscus* (Anura)

**DOI:** 10.1007/s10646-026-03140-6

**Published:** 2026-08-20

**Authors:** Wadson Rodrigues Rezende, Bruno Serra de Lacerda Valverde, Lia Raquel de Souza Santos, Lilian Franco-Belussi, Classius De Oliveira

**Affiliations:** 1 Laboratory of Comparative Anatomy, Department of Biology, UNESP: Universidade Estadual Paulista Julio de Mesquita Filho, São José do Rio Preto, São Paulo, Brazil; 2 Laboratório de Ecotoxicologia e Sistemática Animal (EcotoxSA), Instituto Federal Goiano, Rio Verde, Goiás, Brazil; 3 Laboratory of Experimental Pathology, Institute of Biosciences, Federal University of Mato Grosso Do Sul, Mato Grosso Do Sul, Campo Grande, Brazil

**Keywords:** Agriculture, Amphibians, Spleen, Blood, Testis, Urbanization

## Abstract

Environmental changes caused by anthropogenic activities are accompanied by a multitude of chemical compounds and stressors, which exert significant pressure on native anuran communities. Many of these substances are endocrine disruptors, which interfere with the organism’s physiology, particularly the immune and reproductive systems. We used 49 male *Leptodactylus fuscus* collected in agricultural areas (soybean and sugarcane) and urban environments in the states of Goiás and São Paulo (Brazil) and compared them with each other and with minimally affected specimens from an environmental conservation unit (Emas National Park – Goiás). In the anthropized areas, there was an increase in the number of lymphocytes and neutrophils. The spleen of specimens collected in the different anthropized environments showed a decrease in white pulp, an increase in red pulp, an increase in mast cells, and a change in melanin pigmentation. In animals exposed to greater environmental disturbance, there was a tendency for an increase in the area occupied by spermatogonia, spermatocytes, spermatids, and spermatozoa. These results indicate that landscape anthropization is associated with multi-systemic physiological modifications, highlighting the role of tissue biomarkers in monitoring wild populations of *L. fuscus* under environmental stress.

## Introduction

Activities such as agriculture, agribusiness, and urban expansion place strong pressures on native organism communities, causing habitat fragmentation, water resource occupation, and the generation of large amounts of waste, which can be released directly into the environment without proper treatment (Ehrenfeld 2000; Foley et al. 2005; Grimm et al. 2008; Nellemann et al. 2009; Schiesari and Grillitsch 2011; Hansen et al. 2012; Newbold et al. 2015; Newbold 2018; Beckmann et al. 2019; Cordier et al. 2021; Pongratz et al. 2021). Each of these anthropogenic scenarios presents distinct characteristics. Agricultural activities, for example, utilize large tracts of land (CONAB 2022; IBGE 2022; MapBiomas 2023), alter soil properties, increase greenhouse gas emissions (Pongratz et al. 2021), and release a wide range of chemical compounds (agrochemicals) that are used for soil correction and fertilization and to control agricultural pests (Clay 2004; Cunha et al. 2008; Schiesari and Grillitsch 2011). Agroindustrial activities, especially the sugar and alcohol industries, generate numerous residues associated with the production chain, such as combustion gases, washing effluents from various processes, lubricating oils from machinery, and settled solids (Filoso et al. 2015; Christofoletti et al. 2013). In addition, sugarcane cultivation generates byproducts such as vinasse (Rebelato et al. 2013), a fertilizer rich in organic matter, phosphorus, potassium, and nitrogen. These products can modify the ionic composition of the soil and contaminate nearby water bodies (Filoso et al. 2015; Fuess et al. 2018, 2021; Acayaba et al. 2021). Similarly, soybean cultivation has an intensive and continuous application of agrochemicals. This includes broad-spectrum herbicides (predominantly glyphosate-based formulations), systemic insecticides (such as pyrethroids, organophosphates, and neonicotinoids), and fungicides, alongside synthetic fertilizers (Schiesari et al. 2013). The runoff and leaching of these xenobiotics frequently contaminate adjacent aquatic and semi-aquatic systems, imposing severe ecotoxicological stress on native non-target organisms, particularly amphibians (Relyea 2005; Peltzer et al. 2008; Brodeur et al. 2011).

Urbanization is complex because there are multiple sources of waste, such as residential and industrial water discharges, storm water runoff, accidental spills, and direct dumping of solid waste (Mackintosh and Davis 2013). The waste includes a variety of compounds, such as pharmaceuticals, hormones, cosmetics (Tijani et al. 2016; Da Silva et al. 2018; Matthiessen et al. 2018), heavy metals (Esbaugh et al. 2018; Gosset et al. 2020), disinfectants and residues from incomplete combustion of fossil fuels (Juhasz and Naidu 2000; Zakaria et al. 2002; Srogi 2007; Richardson 2008; Bukowska et al. 2022). Many of these chemicals are considered potential endocrine disruptors (EDs) which can alter the physiology of exposed organisms (Kolpin et al. 2002; Srogi 2007; Richardson 2008; Chang et al. 2009; Da Silva et al. 2018; Matthiessen et al. 2018; Esbaugh et al. 2018). Anurans are particularly vulnerable to these EDs—and consequently serve as excellent bioindicator species—due to their highly permeable skin, biphasic life cycle, and profound reliance on hormonal cues for development and reproduction (Kloas 2002; Hayes et al. 2006, 2010). Because their physiological responses directly reflect the environmental quality of their habitats, specific cellular and tissue alterations in these animals act as efficient biomarkers to estimate the impacts of anthropogenic contaminants. Specifically, EDs have marked effects on anuran immunity by decreasing phagocytic capacity and altering circulating leukocyte populations, resulting in immunomodulation and immunosuppression, leaving anurans susceptible to pathogens (Christin et al. 2004, 2013; Cabagna et al. 2005; Romanova and Egorikhina 2006; Attademo et al. 2014; Pochini and Hoverman 2017; Fanali et al. 2018; McCoy and Peralta 2018; Davis et al. 2020; Bosch et al. 2021). There are also repercussions on the reproductive biology of these animals, with reports of histopathological changes and dysfunction in reproductive organs, especially the testes (McCoy et al. 2008; McDaniel et al. 2008; Çakici 2013, 2015; Moresco et al. 2014; Rojas-Hucks et al. 2019; Rezende et al. 2021; Sluchyk et al. 2021), changes in hormone levels (Poulsen et al. 2015; Campbell et al. 2018; Rojas-Hucks et al. 2019), and altered expression of genes that regulate reproductive processes (Poulsen et al. 2015; Campbell et al. 2018).

Biomarkers represent a very efficient and increasingly used resource to estimate the impacts of contaminants and environmental stress caused mainly by anthropogenic changes. Their potential effects are estimated through the analysis of specific cells and tissues (Livingstone 1993; Fenoglio et al. 2005; Martin et al. 2010). Among these, researchers have investigated immune responses in hematopoietic organs (e.g., the spleen) (Priyadarshani et al. 2015; Çakici 2018; Santana et al. 2021), variations in the leukocyte profile (Priyadarshani et al. 2015; Zhelev et al. 2018; Garcia Neto et al. 2020; Santana et al. 2021), mediators of inflammatory responses (e.g., mast cells and melanomacrophages) (Franco-Belussi et al. 2011, 2021; Provete et al. 2012; De Oliveira et al. 2017; Fanali et al. 2018; Valverde et al. 2022), and reproductive responses through germinal structures (Christin et al. 2004, 2013; Cabagna et al. 2005; Sanchez et al. 2014; McCoy et al. 2017; Fanali et al. 2018).

Brazil has a strong agricultural economy, particularly soybean and sugarcane plantations (743), and is undergoing urban expansion, which involves the occupation and modification of natural habitats used by various organisms. However, despite the growing use of biomarkers in ecotoxicology, there remains a significant research gap regarding how complex, real-world anthropogenic matrices—such as distinct agricultural monocultures versus urban environments—differentially impact native anuran Neotropical species. Most studies still focus on isolated contaminants under controlled conditions, leaving the integrated physiological, immunological, and reproductive trade-offs of wild populations poorly understood. To address this gap, this study aimed to investigate how environments with different patterns of modification (disturbance and contamination) affect the immunological and reproductive parameters of *Leptodactylus fuscus*, an anuran commonly used for biomonitoring.

## Methodology

### Licenses

The collection of specimens was authorized by the Instituto Chico Mendes de Conservação da Biodiversidade (IBAMA-ICMBio, No. 62687-1 and 18573-2). All experimental procedures were approved by the Ethics Committee on Animal Use – IBILCE/UNESP-CSJRP (No. 191/2018) and were performed in accordance with the National Institutes of Health Guide for the Care and Use of Laboratory Animals. Furthermore, all methods and analysis in this study are explicitly reported in accordance with the ARRIVE guidelines (https://arriveguidelines.org).

### Collection sites

**Fig. 1:**
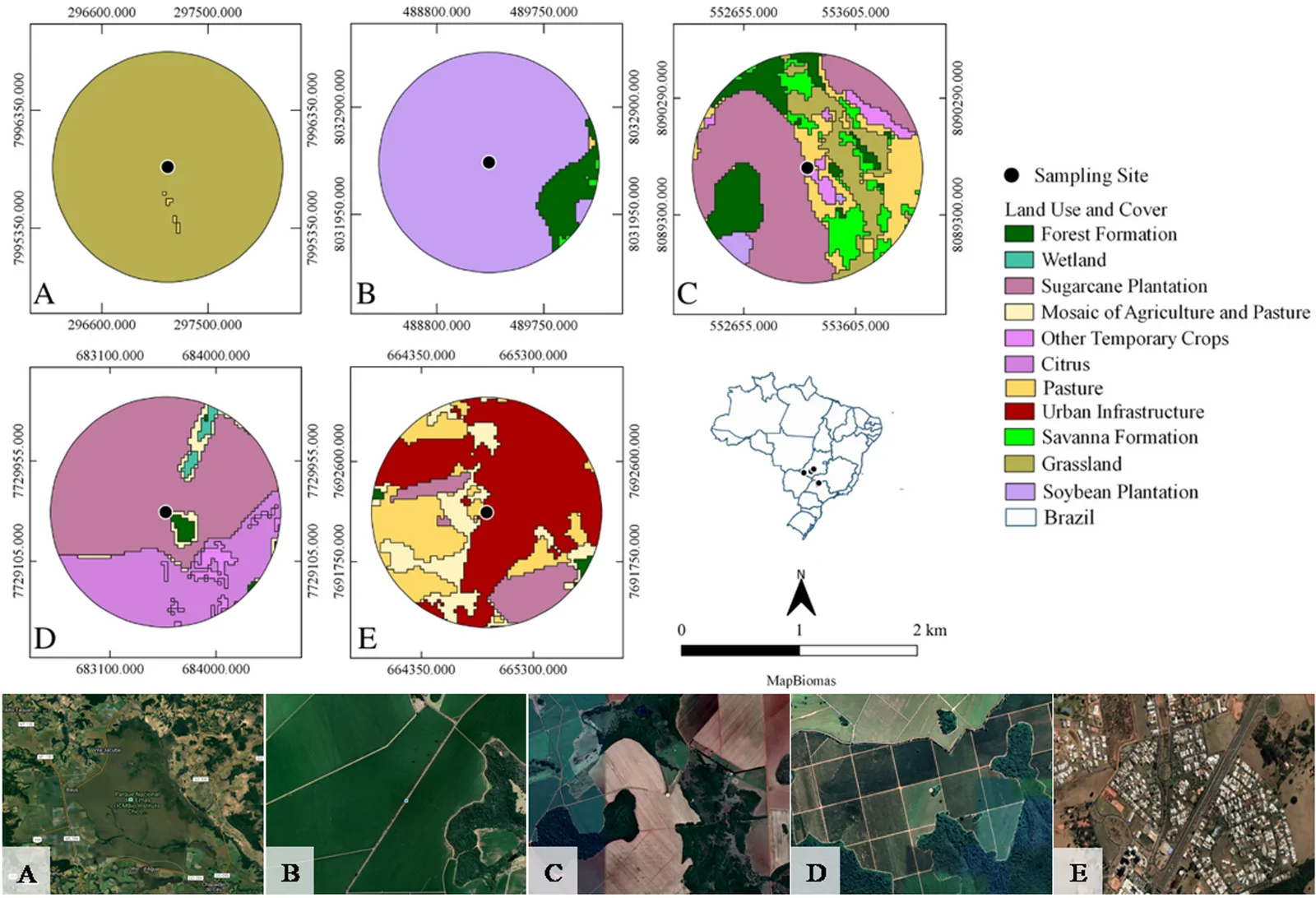
**–** Collection sites and characterization of their environments. A: Conservation Unit (C.Unit); B: Soybean plantation (S-GO); C: Sugarcane plantation (C-GO); D: Sugarcane plantation (C-SP); E: Urban area (U-SP). Classification and characterization according to Google Earth for the years 2018–2020. Radius of 2 km

This study was conducted in the state of Goiás in the Central-West Region of Brazil (the municipalities of Rio Verde and Mineiros), and in the state of São Paulo in the Southeast Region of Brazil (the municipalities of São José do Rio Preto) (Fig. 1). These regions were chosen because both are heavily influenced by human activity due to urbanization as well as agricultural activities. In these locations, four areas with different alteration patterns were selected — a soybean plantation (17°47′44.5″S, 51°06’05.3”W), a sugarcane plantation associated with the sugar and ethanol industries (17°16′38.3″S, 50°29′58.2″W), a sugarcane plantation not associated with the sugar and ethanol industries (20°31′57.3″S, 49°24′53.6″W), and an urban area (20°51′47.9″S, 49°24′53.6″W) — as well as an environmental conservation unit (18°06′59.4″S, 52°55”01.4″W), located in the Emas National Park. In each environment, the percentage of land use matrices was estimated, within a radius of 2 km around the collection points, using the QGIZ software (3.10) (Table 1).

**Table 1:** Proportions of land use matrices within a 2 km radius

Sites	Matrices	%
C.Unit	Grassland	99.7
	Pasture	0.3
S-GO	Soybean Plantation	90.9
	Florest Formation	8.5
	Savanna Formation	0.4
	Pasture	0.2
C-GO	Sugarcane Plantation	40.8
	Pasture	16.3
	Florest Formation	14.2
	Grassland	13.1
	Savanna Formation	9.8
	Other Temporary Crops	4
	Soybean Plantation	1.8
C-SP	Sugarcane Plantation	61.6
	Citrus	28.8
	Mosaic of Agriculture and Pasture	3.6
	Other Temporary Crops	3.6
	Wetland	1.3
	Florest Formation	1.2
U-SP	Urban Infrastructure	51.5
	Pasture	22.2
	Mosaic of Agriculture and Pasture	15.1
	Sugarcane Plantation	10.5
	Florest Formation	0.7

C.Unit: Conservation Unit; S-GO: Soybean plantation; C-GO: Sugarcane plantation; C-SP: Sugarcane plantation; U-SP: Urban area

### Specimen collection

Male *Leptodactylus fuscus* (*n* = 49) vocalizing specimens were collected from 2018 to 2020 in the preserved area and in four anthropogenic settings: the conservation unit (C.Unit, *n* = 10), the soybean plantation (S-GO, *n* = 15), the sugarcane plantations (C-GO, *n* = 7; C-SP, *n* = 10), and the urban environment (U-SP, *n* = 7). The target sample sizes were determined based on historical capture success rates during regional active breeding chorus tracking and were strictly capped in accordance with environmental licensing limits to minimize native population depletion. No animals or extracted tissue samples were excluded from the definitive statistical analyses (total finalized *n* = 49). Vocalizing males were exclusively selected to ensure a robust, homogeneous sample size and to control for baseline physiological variability. This species was chosen as a model organism due to its abundance and wide distribution throughout Brazil, including its occurrence in anthropogenic environments (IUCN 2022). After collection, the specimens were transported to the Animal Biology Laboratory (Instituto Federal Goiano, Rio Verde-GO campus) and the Comparative Anatomy Laboratory (Universidade Estadual Paulista “Júlio de Mesquita Filho”, São José do Rio Preto-SP campus), where they were weighed using a precision analytical balance (0.001 g) and the snout-vent length (SVL) was measured with precision calipers (mm). The biometric data were used to calculate the scaled mass index (SMI), a tool for conservation studies and environmental quality assessment, often used as an indicator of body condition in anurans (Brodeur et al. 2020). The SMI is calculated using the formula:

1 $${\mathrm{SMI}} = {M}_{i} {({L}_{0}/{L}_{i})}^{\mathrm{bSMA}}$$

where *M*_*i*_ represents individual body mass, *L*_*i*_is the individual SVL, *L*_*0*_is the mean SVL of the sampled population, and b is the slope of the major axis regression line (Peig and Green 2009).

### Leukocyte profile

**Fig. 2:**
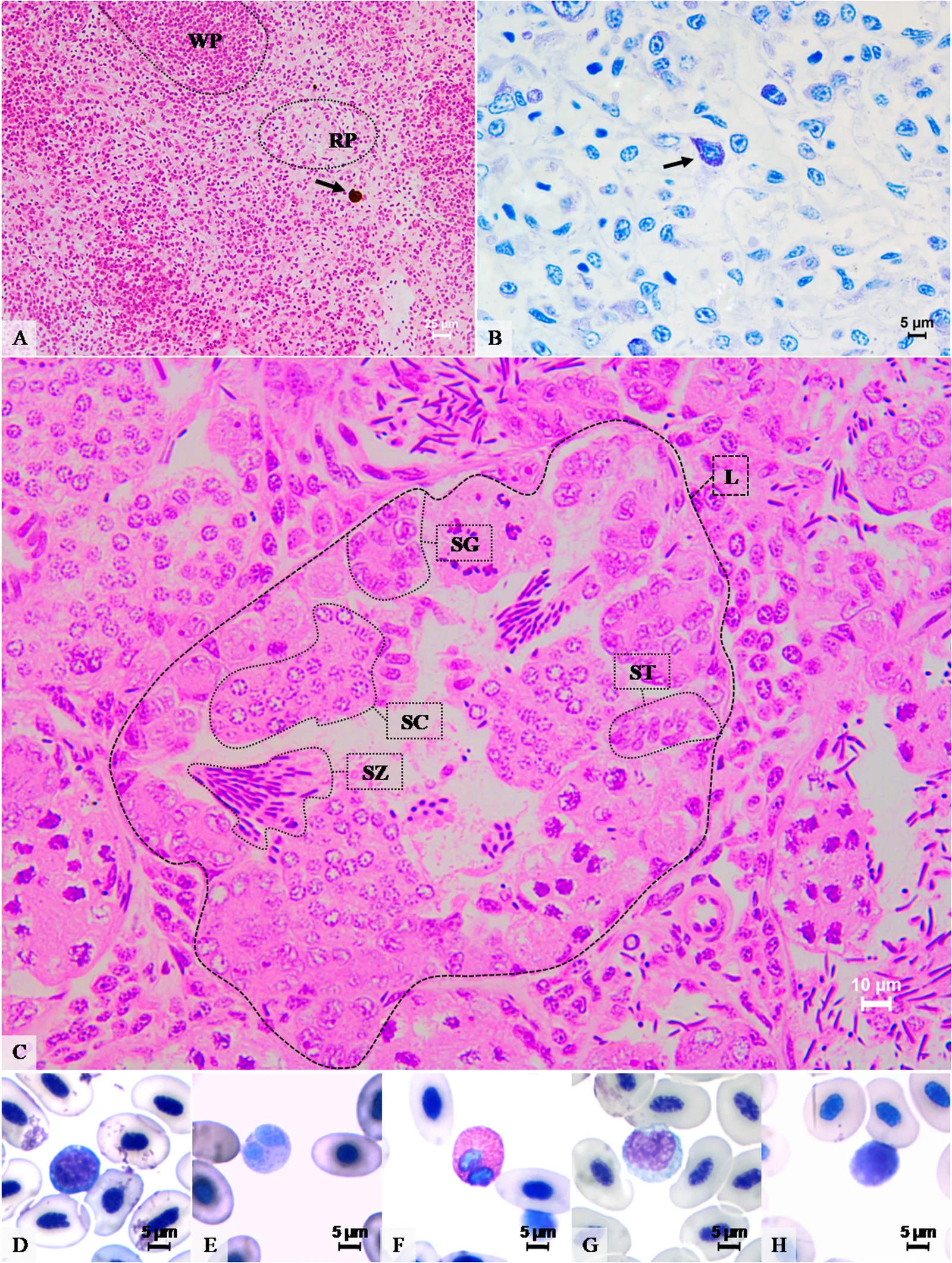
– Histological characterization of the spleen, testis, and leukocytes of *Leptodactylus fuscus*. A (HE – 25 μm): Spleen, red pulp (RP), white pulp (WP), and melanomacrophage (arrow). B (Toluidine blue – 5 μm): Spleen, mast cell (arrow). C (HE – 10 μm): Testis, seminiferous locule (L), spermatogonia (SG), spermatocyte (SC), spermatid (ST), and spermatozoa (SZ). D-H (Giemsa – 5 μm): Leukocyte profile, lymphocytes (D), neutrophil (E), eosinophil (F), monocyte (G), and basophil (H)

Blood samples were collected and placed on histological slides, fixed in methanol, and stained with 7.5% Giemsa solution. For each specimen, the proportion of each cell type (lymphocyte, neutrophil, basophil, monocyte, and eosinophil) in 100 total leukocytes was quantified using a Leica DM4-Blight microscope (with a 100× objective lens), following a published protocol (Franco-Belussi et al. 2018) (Fig. 2D-H).

### Splenic parameters

After collecting biometric data and blood samples, which occurred within 2 h of temporary holding to mitigate confinement stress, the specimens were euthanized via prolonged immersion with 5 g/L benzocaine. Complete death was clinically verified through the confirmed cessation of all motor reflexes and lasting cardiac arrest prior to target tissue harvesting. It was weighed to calculate the Spleen Somatic Index (SSI) using the formula:

2 $${\mathrm{SSI}} = {({M}_{0}/{M}_{i})} \times {100}.$$

where *M*_*o*_ is the spleen mass and *M*_*i*_ is the individual body mass. Subsequently, the spleen was fixed in methacarn solution (60% methanol, 30% chloroform, and 10% acetic acid), dehydrated, and embedded in Historesin (Leica).

#### Splenic volumetric structural density (VSD)

Histological Sect.  (2 μm, cut with a Leica RM2265microtome) were stained with hematoxylin and eosin, photographed under a Leica DM4-B light microscope (with a 40× objective lens), and analyzed using the ImageJ software (version 1.48) (Fig. 2A). A grid was applied to delimit 300 intersections under 10 histological fields by following a protocol adapted from published studies (Freere and Weibel 1967; Reid 1980; Rocha et al. 1997). White pulp, red pulp, blood vessels, and melanomacrophages were quantified. The final density of each structure was calculated using the formula:

3 $${\mathrm{VSD}} {(\%)} = {({\mathrm{IT}} \times {100})}/{\mathrm{Tot}}$$

where It is the number of intersections that contained each structure category, and Tot is the total number of intersections in the image.

#### Mast cell and melanin quantification

For mast cell quantification, 2-µm histological sections were stained with toluidine blue borax solution and analyzed with a Leica DM4-B light microscope (Fig. 2B). The mast cells present in 10 histological sections were counted, and their total area was quantified using the Image-Pro Plus software (version 6.0).

To quantify the melanin present in the melanomacrophages, the sections were stained with hematoxylin and eosin and photographed under a Leica DM4-B light microscope (with a 25×objective lens). The melanin area present in 25 histological fields was determinate following a protocol adapted from Santos et al. 2014.

### Reproductive parameters

The testes were removed and weighed to calculate the gonadosomatic index (GSI) using the formula:

4 $${\mathrm{GSI}} = {({M}_{0}/{M}_{i})} \times {100}$$

where *M*_*o*_ is the testis mass and *M*_*i*_ is the individual body mass. Subsequently, the testes were fixed in methacarn solution, dehydrated, and embedded in Historesin. For analysis of reproductive traits, 2-µm histological sections were stained with hematoxylin-eosin, photographed under a Leica DM4-B light microscope (with a 20× objective lens), and analyzed using Image-Pro Plus software. For each specimen, 100 seminiferous locules were analyzed to determinethe locular area and the area of spermatogenic cysts, categorized according to Santos and Oliveira (2008) as spermatogonia, spermatocytes, spermatids, and spermatozoa. These measurements were obtained by circumscribing the seminiferous units and germinal cysts (Fig. 2C).

Mast cells were also quantified in the testes, using the same methodology as for the spleen (see Sect. Mast cell and melanin quantification).

### Statistical analysis

To estimate changes in the biometric, immunological, and reproductive parameters, a pairwise comparison was performed between the anthropized areas and the conservation unit. A linear model was used for the biometric data (Mass, SVL, SMI, SSI, and SGI). The model assumptions—variance distribution and homogeneity—were tested using diagnostic plots (sjplot package). The spleen melanin area was also subjected to the same model, but the data were log transformed to ensure normal distribution and homogeneity of the residuals. For data on the VSD and mast cell frequency (spleen/testis), a generalized linear model with binomial distribution was used. For the leukocyte profile and testicular morphometric parameters, an analysis of variance model was used. To eliminate potential investigator assessment bias, all differential leukocyte profiling, splenic structural intersection counts, and spermatogenic cyst area measurements were conducted in a strictly blinded manner, utilizing randomly coded slide labels so that the microscopic evaluator remained unaware of each individual specimen’s geographic origin matrix throughout the quantitative phase.

## Results

### Biometric parameters

**Fig. 3:**
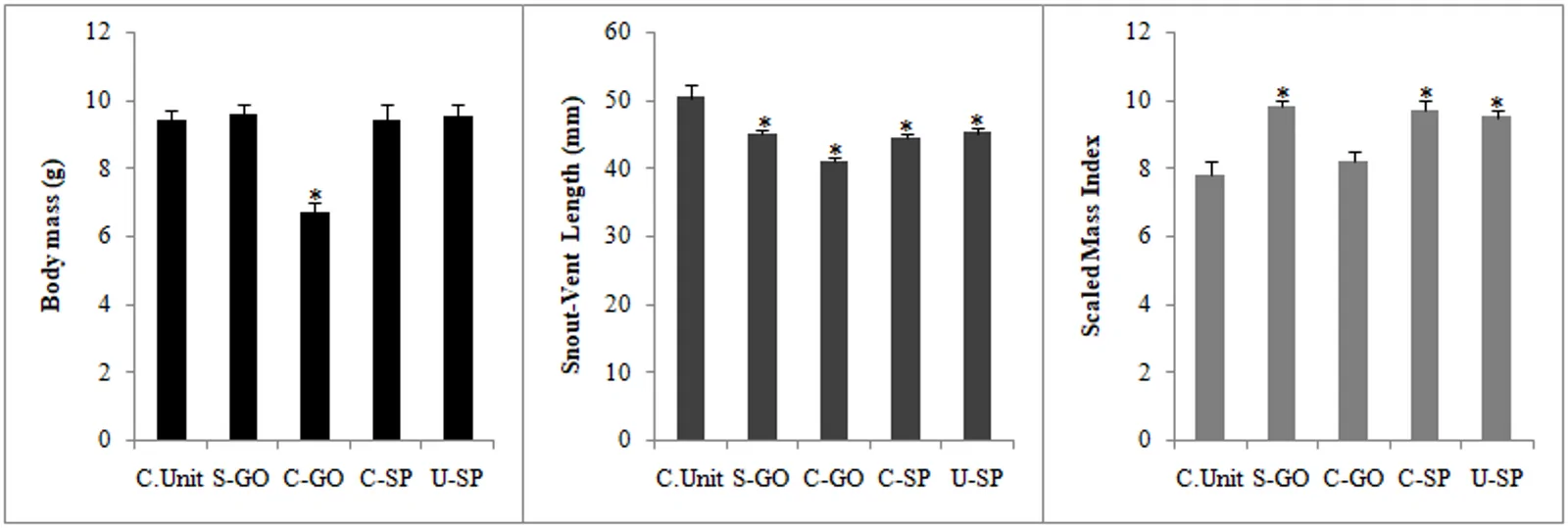
**–** Biometric data (mean ± SE) of *Leptodactylus fuscus*. ‘*’ Significant difference (*p* < 0.05) in relation to the Conservation Unit (C.Unit). S-GO: Soybean plantation; C-GO: Sugarcane plantation; C-SP: Sugarcane plantation; U-SP: Urban area

Overall, specimens from C.Unit had a mean weight of 9.4 ± 0.3 g, a SVL of 50.4 ± 2.0 mm, and an SMI of 7.8 ± 0.4. The specimens from the anthropized areas showed notable changes in these parameters: there was a decrease in weight in specimens from C-GO (6.7 ± 0.3 g); a decrease in the SVL in specimens from U-SP (45.2 ± 0.6 mm), S-GO (45.0 ± 0.6 mm), C-SP (44.4 ± 0.7 mm), and C-GO (41.0 ± 0.7 mm); and an increase in the SMI in specimens from S-GO (9.8 ± 0.2), C-SP (9.7 ± 0.3), and U-SP (9.5 ± 0.2) (Fig. 3).

### Leukocyte profile

**Fig. 4:**
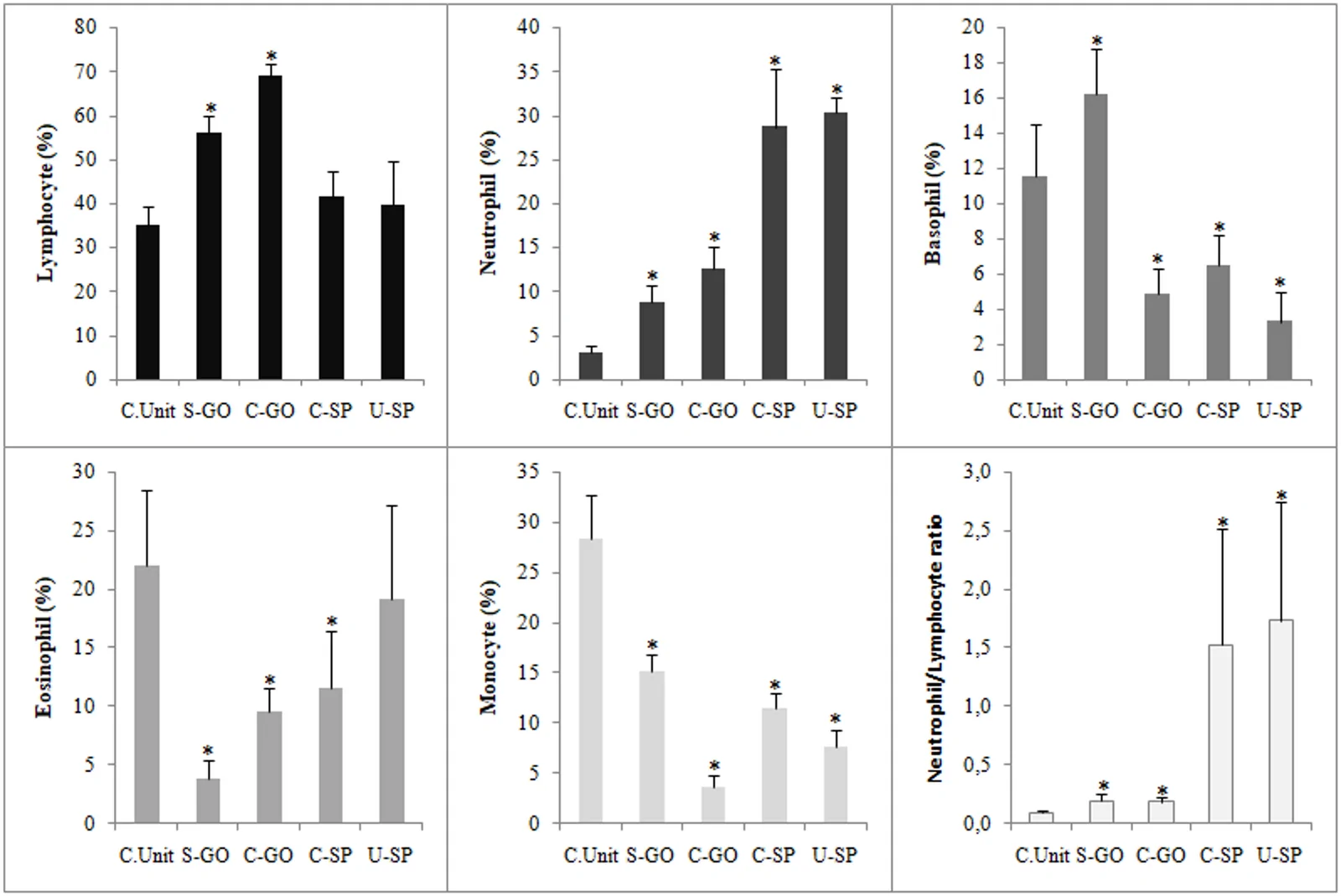
– Proportion of leukocytes in *Leptodactylus fuscus* (mean ± SE). ‘*’ Significant difference (*p* < 0.05) in relation to the Conservation Unit (C.Unit). S-GO: Soybean plantation; C-GO: Sugarcane plantation; C-SP: Sugarcane plantation; U-SP: Urban area

In blood samples obtain from the C.Unit specimens, considered a “normal standard” or control, lymphocytes were the predominant cell type (35.1 ± 4.3%), followed by monocytes (28.4 ± 4.2%), eosinophils (22.0 ± 6.4%), basophils (11.5 ± 3.0%), and neutrophils (3.0 ± 0.8%). Lymphocytes were also predominant in the specimens from the anthropized areas, although the proportion was increased in specimens from C-GO (69.3 ± 2.8%) and S-GO (56.0 ± 3.9%). There was also an increase in neutrophils (AU-SP: 30.3 ± 1.7%; C-SP: 28.7% ± 6.6%; C-GO: 12.6% ± 2.5%; S-GO: 8.7% ± 2.0%) and basophils (S-GO: 16.2% ± 2.6%) in some anthropized environments. On the other hand, specimens from some environments showed a decrease in basophils (U-SP: 3.3 ± 1.7%; C-GO: 4.9 ± 1.4%; C-SP: 6.5 ± 1.7%), eosinophils (S-GO: 3.9 ± 1.5%; C-GO: 9.6 ± 1.9%; C-SP: 11.6 ± 4.9%), and monocytes (C-GO: 3.7 ± 1.1%; U-SP: 7.7 ± 1.7%; C-SP: 11.5 ± 1.5%; S-GO: 15.2 ± 1.7%) (Fig. 4).

### Splenic parameters

**Fig. 5:**
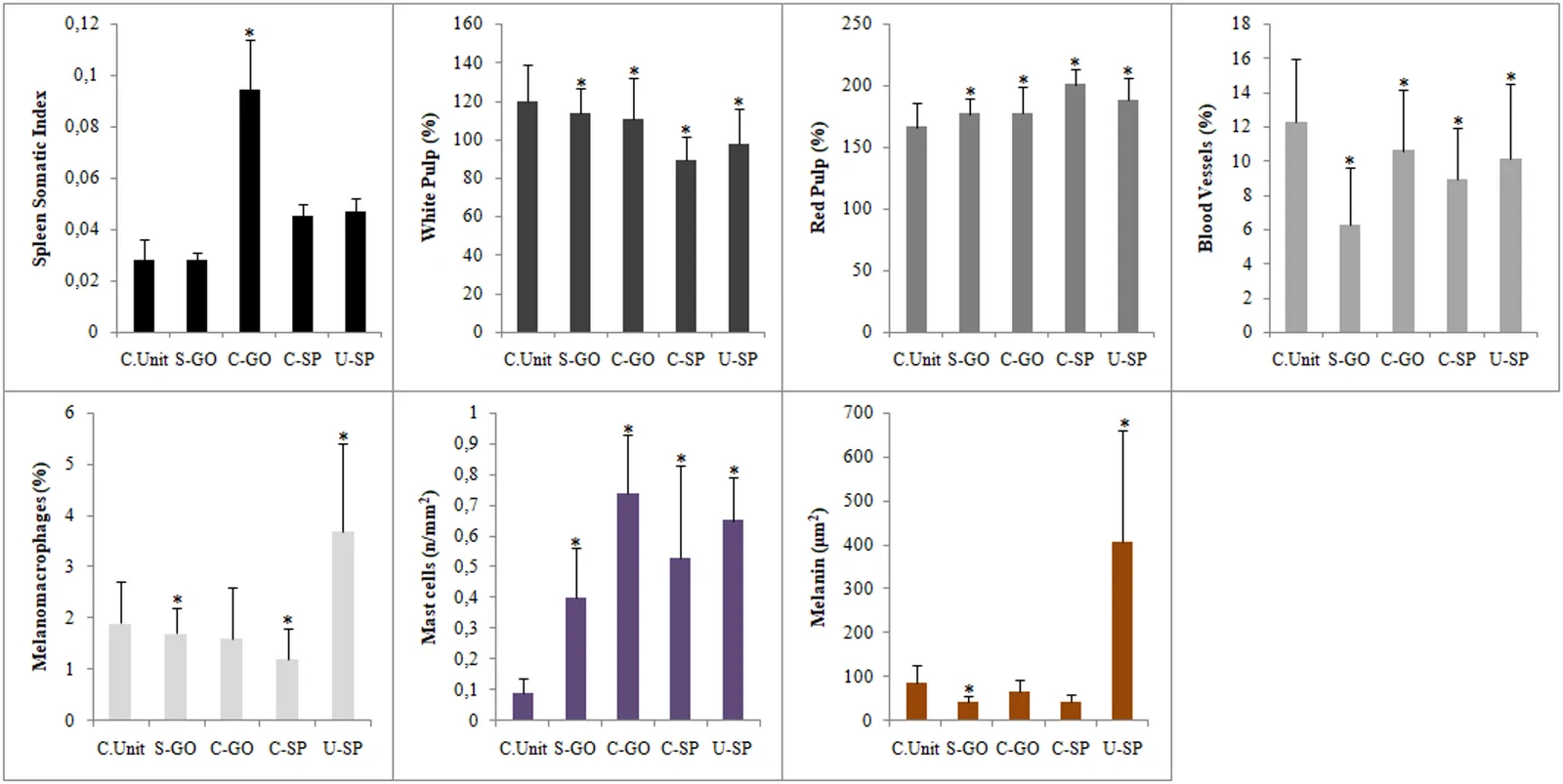
**–** Splenic parameters of *Leptodactylus fuscus* (mean ± SE). ‘*’ Significant difference (*p* < 0.05) in relation to the Conservation Unit (C.Unit). S-GO: Soybean plantation; C-GO: Sugarcane plantation; C-SP: Sugarcane plantation; U-SP: Urban area

The spleen of the C.Unit specimens had an SSI of 0.028 ± 0.008 and a majority of red pulp (166.3 ± 19.5 intersections), followed by white pulp (119.5 ± 19.3 intersections), blood vessels (12.3 ± 3.7 intersections), and melanomacrophages (1.9 ± 0.8 intersections). Furthermore, these specimens presented 0.09 ± 0.05 mast cells/mm² and a melanin area of 85.57 ± 39.7 μm² (Fig. 5).

The SSI was increased in the specimens collected in C-GO (0.094 ± 0.020). All evaluated splenic components showed significant changes in the specimens from anthropized areas, with an increase in red pulp (C-SP: 200.8 ± 12.7; U-SP: 188.3 ± 17.4; C-GO: 177.8 ± 21.1; S-GO: 177.0 ± 13.0 intersections) and a decrease in white pulp (C-SP: 89.2 ± 12.6; U-SP: 97.9 ± 18.2; C-GO: 110.6 ± 21.2; S-GO: 113.6 ± 13.0 intersections) and blood vessels (S-GO: 6.3 ± 3.3; C-SP: 8.9 ± 3.0; U-SP: 10.1 ± 4.4; C-GO: 10.6 ± 3.6 intersections). The specimens from C-SP and S-GO exhibited a decrease in melanomacrophages (1.2 ± 0.6 and 1.7 ± 0.5 intersections, respectively), while U-SP showed an increase (3.7 ± 1.7 intersections) (Figs. 5). The frequency of mast cells and the melanin area also showed changes due to the anthropic environment, with a generalized increase in mast cells (C-GO: 0.74 ± 0.19; U-SP: 0.65 ± 0.14; C-SP: 0.53 ± 0.30; S-GO: 0.40 ± 0.16 mast cells/mm²), while melanin increased in specimens from U-SP (408.19 ± 251.4 μm²) and decreased in specimens from S-GO (41.69 ± 15.65 μm²) (Fig. 5).

### Reproductive parameters

**Fig. 6:**
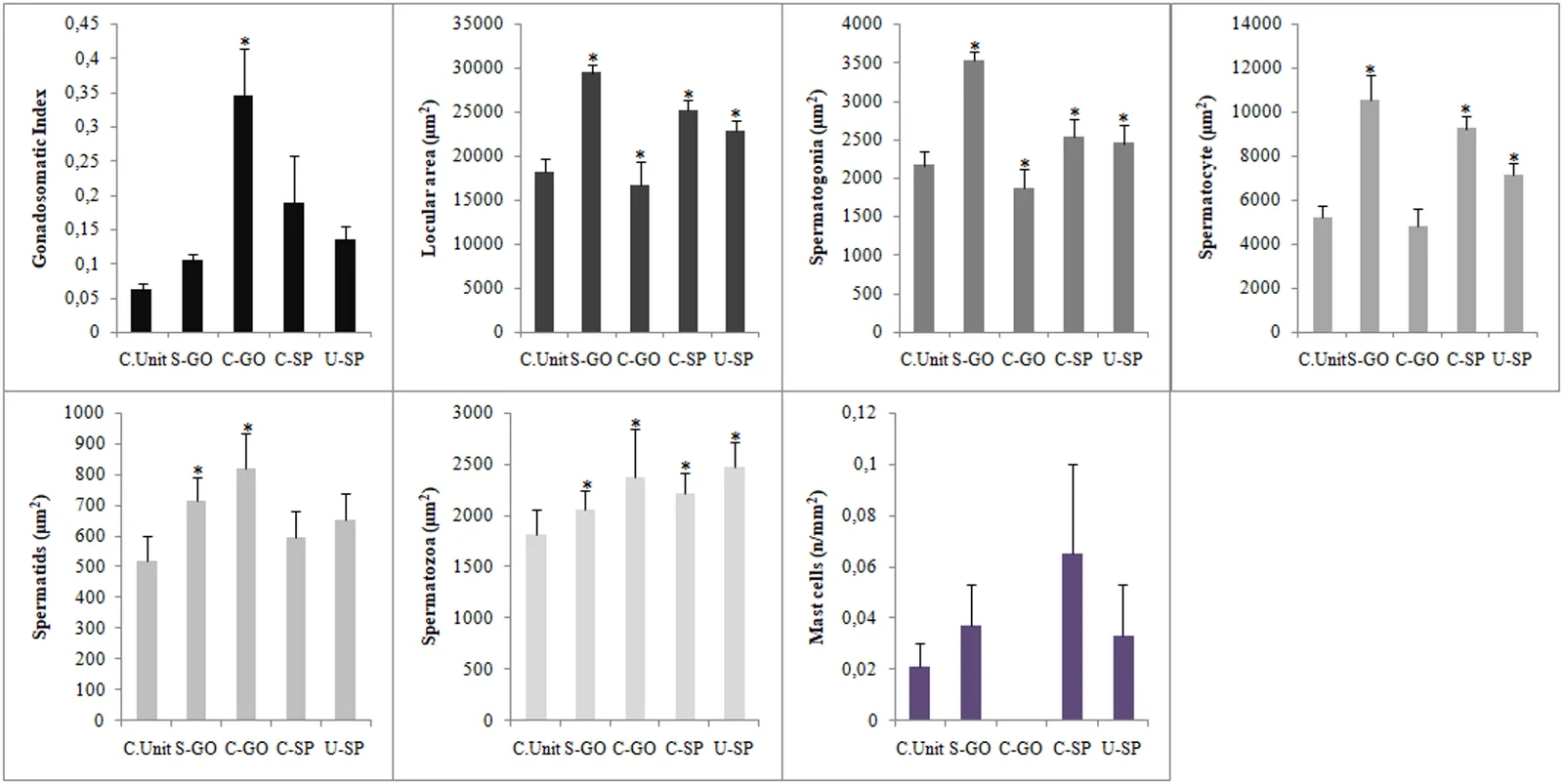
– Repoductive parameters of *Leptodactylus fuscus* (mean ± SE). ‘*’ Significant difference (*p* < 0.05) in relation to the Conservation Unit (C.Unit). S-GO: Soybean plantation; C-GO: Sugarcane plantation; C-SP: Sugarcane plantation; U-SP: Urban area

The C.Unit specimens had a mean GSI of 0.063 ± 0.007 and the mean seminiferous locular area was 18,174 ± 2,678 μm², with the following germ cells: spermatogonia (2,163 ± 178 μm²), spermatocytes (5,186 ± 556 μm²), spermatids (521 ± 78 μm²), and spermatozoa (1,815 ± 243 μm²). In addition, these specimens presented 0.021 ± 0.009 mast cells/mm² (Fig. 6).

The GSI increased significantly in specimens from C-GO (0.344 ± 0.071). The seminiferous locular area increased in specimens from S-GO (29451 ± 996 μm²), C-SP (25182 ± 1249 μm²), and U-SP (22824 ± 1284 μm²), and decreased in specimens from C-GO (16712 ± 2672 μm²). In general, spermatogonia (S-GO: 3533 ± 111; C-SP: 2538 ± 235; U-SP: 2451 ± 243 μm²), spermatocytes (S-GO: 10553 ± 1125; C-SP: 9234 ± 607; U-SP: 7166 ± 488 μm²), spermatids (C-GO: 821 ± 112; S-GO: 714 ± 78), and spermatozoa (U-SP: 2469 ± 240; C-GO: 2371 ± 468; C-SP: 2209 ± 208; S-GO: 2060 ± 176 μm²) showed an increase in area in specimens collected in anthropized areas. There was a decrease in spermatogonia in specimens from C-GO (1865 ± 256 μm²) (Fig. 6) compared to C.UNIT.

Regarding mast cells, there were no significant differences in specimens collected in any of the anthropized environments (Fig. 6).

## Discussion

### Biometric parameters and body condition

Biometric indices are tools that provide information about the body condition displayed by animals; they can reflect their health and predict the effects of anthropogenic pressures. Decreased body condition in specimens from anthropogenic areas has been associated with altered environments and environmental pollutants, such as herbicides (Delgado-Acevedo and Restrepo 2008; Karlsson et al. 2021), demonstrating that anthropogenic disturbances decrease body size in anurans. Although body size is strongly correlated with fertility in anurans, this attribute is strictly representative of females—where size dictates clutch volume. Consequently, male body size does not directly influence primary fertility rates (Fan et al. 2013; Ding et al. 2019). While incorporating a statistically robust cohort of females would be highly valuable to fully understand population viability, our data demonstrate that the observed reduction in male SVL does not, by itself, represent a direct decline in the primary reproductive output of these populations. However, the lack of female reproductive endpoints represents an important limitation in fully translating these individual fitness traits into long-term population-level consequences.

Body condition indicators, such as the SMI, have been used as a minimally invasive tool to estimate the health, energy reserves, and fitness of animals (Stevenson and Woods Jr, 2006; Peig and Green 2010; MacCracken and Stebbings 2012; Brodeur et al. 2020). In general, anthropized environments are expected to exert negative pressure on body condition indicators (i.e., decrease their values), as in *Leptodactylus latinasus* and *Rhinella fernandezae* collected in soybean fields (Brodeur et al. 2011), and *Dendropsophus ebraccatus* from highly modified environments (Matías-Ferrer and Escalante 2015). However, some species tend to benefit from altered environments. Iglesias-Carrasco et al. (2017) reported that amphibians living in urban environments showed an increase in these body mass indexes, possibly due to the adaptation of the organisms to unconventional food sources, such as new exotic invertebrate communities. Because *L. fuscus* is a generalist species in terms of its feeding habits (Santana et al. 2019), we assume that the higher mean SMI we found is related to a greater energy reserve, likely the result of dietary adaptation. Concurrently, immune responses require energy (Demas 2004) and thus increase metabolic rates (Sherman and Stephens 1998; Gardner et al. 2020). This would account for the greater energy reserve (expressed by the SMI) in our specimens: It is a response mechanism to meet the immunological demands exerted by anthropogenic pressure. We observed this immunological demand in both the leukocyte profile and the splenic parameters of *L. fuscus*.

### Leukocyte profile

Changes in hematological parameters reflect an organism’s adaptive response to the influence of a complex set of environmental factors, and provide a way to estimate the effect of anthropogenic pressures and their pollutants (Romanova and Egorikhina 2006). Among these parameters, the leukocyte profile has been a useful tool for estimating the effects of stressors on anurans (Zheleve and Popgeotgiev 2021) and the degree of immunological challenge to which these animals are subjected (Romanova and Egorikhina 2006).

Lymphocytes and neutrophils are the most abundant and relevant cell types for characterizing the leukogram of anurans (Franco-Belussi et al. 2022). Neutrophils are granulocytes with phagocytic action; they act in the innate immune response and in inflammatory processes (Davis et al. 2008; Shutler et al. 2009). Lymphocytes represent an organism’s investment in the adaptive immune response, and also act in rapid systemic responses (Fournier et al. 2005; Shutler et al. 2009). Changes in these two cell types are considered a sensitive and direct indicator of the effect of stress caused by anthropogenic action (Romanova et al. 2019; Garcia Neto et al. 2020). The most representative picture of the effect of modified environments and chemical pollutants is an increase in the number of neutrophils (neutrophilia) (neutrophilia) and a decrease in the number of leukocytes (leukopenia) (Davis et al. 2008; Assis et al. 2017; Romanova et al. 2019). However, we observed an increase in these two cell types in specimens collected in anthropized areas. Specimens from altered environments present high levels of glucocorticoid hormones (Janin et al. 2011; Rollins-Smith 2017; Davis et al. 2020), which activate neutrophils and increase their numbers (Falso et al. 2015; Assis et al. 2017). Granulocyte leukocytes, such as neutrophils, release pro-inflammatory factors (Fournier et al. 2005; Davis et al. 2008; Romanova et al. 2019), and environmental pollution can cause anurans to produce more inflammatory mediators (represented by neutrophil activity), which consequently leads to the increase in and migration of lymphocytes (Romanova et al. 2019).

Basophils, eosinophils, and monocytes are also immune cells. Basophils act in inflammatory responses by modulating the defense against parasites and allergens (Falcone et al. 2006; Davis et al. 2008); eosinophils generally act in parasitic infections (Davis et al. 2008; Campbell 2012); and monocytes act against bacteria and helminths (Davis et al. 2008; Marcogliese et al. 2009). Although these cells are less numerous than other immune cells, their combined activity ensures an efficient immune response. In our study, we observed a general decrease in basophils, eosinophils, and monocytes, with only a specific increase in basophils in specimens from S-GO. Anurans in agricultural environments present a greater abundance and intensity of helminth infections (Portela et al. 2020), which may be associated with a decrease in the number of eosinophils and basophils, increasing the susceptibility of these animals to parasites. Some agrochemicals also alter these immune cells: The insecticide chlorpyrifos decreases basophils in *Odontophrynus carvalhoi* (Silva et al. 2020), while atrazine, malathion, and acevaltrate suppress circulating eosinophils in *Rana sylvatica*, leading to increased trematode infections (Kiesecker 2002). Studies have also reported a decrease in monocytes in environments with high agricultural activity or high levels of anthropogenic disturbance (Marcogliese et al. 2009; Garcia Neto et al. 2020). When these cells migrate to sites of inflammation, they differentiate into macrophages (Grogan et al. 2018). Hypothetically, the reduction in this cell type may reflect the phagocytic response of these animals.

### Splenic parameters

The anuran spleen functions as a primary lymphoid organ (the site of lymphopoiesis) and primarily as a secondary lymphoid organ (the site of antigen presentation, activation, and expansion of T and B lymphocytes) (Grogan et al. 2018). Considering that this organ is a target of direct and indirect toxicity (Suttie 2006), the effects of different anthropogenic pressures can affect its morphology in different ways. Pesticides such as carbaryl cause degeneration of the splenic structure (Çakici 2018), while a mixture of pesticides decreases the number of splenocytes (Christin et al. 2004), and agricultural areas reduce the viability of these cells (Christin et al. 2013). White pulp in the spleen is formed by an aggregate of lymphoid tissue with lymphopoietic activity (Du Pasquier et al. 1989; Mebius and Kraal 2005). We found a lower proportion of white pulp in *L. fuscus* collected in anthropized areas. Linzey et al. (2003) also reported a decrease in while pulp in *Bufo marinus* naturally exposed to agrochemicals (p, p’-dichlorodiphenyl dichloroethylene [p-p’DDE]) and heavy metals (cadmium, chromium, copper, and zinc). Riaz et al. (2021) observed that frogs exposed to the insecticide alpha-cypermethrin exhibited an increased abundance of white pulp. This demonstrates that environmental pollutants can both suppress and stimulate the immune response (immunomodulation) or even generate abnormal responses such as autoimmunity (Fournier et al. 2005). This may affect the morphology of the spleenand thus impair its functions (Çakici 2018), making anurans more susceptible to disease.

Another important function of the spleen is hemocateresis—the destruction of old and/or damaged erythrocytes—performed in red pulp (Suttie 2006; Ross and Pawlina 2018). The greater proportion of red pulp we observed may reflect increased activity, because anurans in disturbed environments tend to have a higher rate of erythrocyte nuclear abnormalities (Şişman et al. 2015; Borges et al. 2019; Assis et al. 2021; Zhelev et al. 2022).

Hemocateresis is also attributed to melanomacrophages that reside in red pulp (Agius and Roberts 2003; Franco-Belussi and De Oliveira 2016). The phagocytic activity of these cells removes blood cells and recycles iron through the degradation of hemoglobin (Agius and Agbede 1984). Melanin is also present in these cells as part of the immune response against environmental contaminants (De Oliveira and Franco-Belussi 2012; Franco-Belussi and De Oliveira 2016; De Oliveira et al. 2017). The lower percentage of melanomacrophages observed in specimens from C-SP and S-GO may be related to a melanin response due to long-term exposure, where high levels of chemical compounds accumulate in this pigment, to the point of causing cell degeneration (Larsson 1993). Compounds such as benzopyrene, which is abundant in urban environments (Bukowska et al. 2022), and herbicides and agricultural fertilizers—such as atrazine and phosphate, respectively—decrease the melanin area in hepatic melanomacrophages (Rohr et al. 2008; Fanali et al. 2018; Valverde et al. 2022).This effect has also been reported for animals found in soybean plantations (Assis et al. 2021). In contrast, the increase in melanin in specimens collected from AU-SP may be related to the protective activity of melanin against environmental contaminants. De Oliveira et al. (2017) also reported this increase in anurans collected in soybean and corn fields, as well as in anurans exposed to surfactants such as linear alkylbenzene sulfonate (LAS) (Franco-Belussi et al. 2021), a compound from urban and industrial waste (Oliveira et al. 2010).

There is a close anatomical relationship between melanomacrophages and mast cells in anurans (Chieffi-Baccari et al. 1998), which are also found in red pulp (Franco-Belussi and De Oliveira 2016). Mast cells are involved in the inflammatory process (Chieffi-Baccari et al. 2011), and an increase in their frequency has been reported in species exposed to benzopyrene (Fanali et al. 2018; Valverde et al. 2022) and surfactants (Franco-Belussi et al. 2021). Our results also showed an increase in mast cells, indicative of an inflammatory process in the tissue (Da Silva et al. 2011), a possible response to stress exerted by the environment and the compounds present there.

The spleen is closely linked to the circulatory system (Abdul-Majeed 2008; Akat 2018), given the role of this organ in blood cell retention, hematopoiesis, and immunity (Mebius and Kraus 2005; Abdul-Majeed 2008; Çakici 2018). Given that immune responses are systemic and interrelated (Romanova and Egorikhina 2006), we can assume that changes in the splenic structure (especially in white pulp) may affect leukocyte populations—as observed in the leukocyte profile of *L. fuscus*—which constitutes a generalized alteration in the immune response in specimens residing in environments subject to anthropogenic alteration.

### Reproductive parameters

Testicular size is an effective marker of the reproductive status, so reproductive timing and development can be predicted based on indices such as the GSI (Othman et al. 2011). Although we did not observe changes in the GSI, there are other ways to assess the reproductive parameters of a population, such as changes in the proportion of the germinal epithelium and various morphological analyses (Ramm and Schärer 2014; Leite et al. 2015; Rezende et al. 2021). It is known that spermatogenesis is regulated by androgenic hormones (Rastogi et al. 2011), and many chemical pollutants are EDs with androgenic or antiandrogenic activity (Kloas and Lutz 2006), such as the herbicide linuron, which causes an increase in seminiferous locules and the number of spermatogonia (Orton et al. 2018), a masculinizing effect. In Brazil, linuron has historically been registered and utilized as a selective pre-emergence herbicide to control broadleaf weeds, particularly in soybean cultivation. Although its commercial use in the country has recently faced strict prohibitions due to its severe toxicological profile (ANVISA 2022), this compound was actively applied during the specific timeframe of our field collections (2018–2020). Therefore, the historical footprint and persistence of linuron, along with other urea-derivative herbicides extensively used in both soybean and sugarcane management, represent a realistic exposure scenario that potentially contributes to the endocrine and germinal alterations observed in wild *L. fuscus* populations (Albuquerque et al. 2016; Pignati et al. 2017). While direct chemical quantification was not performed in our study, these historical management practices provide a plausible contextual framework for the observed tissue disruptions. Similar to the trend we observed for an increase in most germinal cysts, anurans collected in agricultural areas (soybean and rice plantations) also exhibited a larger area of initial germinal cysts (i.e., spermatogonia and spermatocytes) and sperm, compared with specimens collected in preserved environments (Curi et al. 2021; Rezende et al. 2021). Although habitat alterations can cause significant losses and extinctions of species that are considered specialists, generalists may ultimately benefit from these drastic changes (Catenazzi 2015; Pyron 2018). Anthropogenic environments can lead species to invest more in reproduction to compensate for decreased life expectancy (Zamora-Camacho and Comas 2017), as individual reproductive success and population viability can be increased if exposure to pollutants during the reproductive period does not compromise reproduction overall (Bókony et al. 2018). Thus, despite the initially detrimental effects of pollutants on non-target organisms, populations can evolve in response to these stressors, in some cases acquiring resistance to these compounds (Hua et al. 2015).

Even though an increase in reproductive parameters may seem favorable at first glance, they are not necessarily so. Our results provide only quantitative data on the germinal epithelium, while researchers have reported the negative effect of disruptive compounds and altered environments on fertility and gamete viability. Anthropogenic environments—such as cities, industrial sites, and areas contaminated with heavy metals—lead to low sperm counts and marked changes in sperm parameters, including decreased motility and an increase in sperm deformities (Guo et al. 2018; Sluchyk et al. 2021). While our findings provide clear evidence of physiological trade-offs in males, there is significant scope for future ecotoxicological studies to include female specimens. Females have distinct energy allocation strategies due to the high costs of oogenesis and investigating how anthropogenic matrices affect their specific immune-reproductive trade-offs will be essential to fully understand population-level impacts.

## Conclusion

In conclusion, anthropized landscapes—specifically soybean, sugarcane, and urban matrices—exert significant multi-systemic pressures on wild *Leptodactylus fuscus* populations, inducing chronic immune-inflammatory stress characterized by leukocyte alterations and splenic lymphoid depletion, alongside an adaptive reproductive trade-off marked by testicular germinal epithelium alteration. Although these field data offer valuable integrative insights, important limitations must be fully recognized. First, the exclusive sampling of adult vocalizing males prevents a comprehensive assessment of maternal contaminant transfer or impacts on female primary fecundity traits. Second, our exposure assessment relied strictly on land-use matrices as landscape proxies rather than direct environmental, tissue, or bioaccumulation chemical quantification. Therefore, future research should prioritize the inclusion of robust female cohorts to evaluate maternal contaminant transfer and primary fecundity traits, while coupling histomorphometric biomarkers with direct tissue residue analyses and qualitative gamete assays to fully understand the long-term demographic viability of Neotropical amphibians facing ongoing habitat transformation.

## Data Availability

The data that support the findings of this study are available from the corresponding author.
